# Successful adoptive transfer and *in vivo* expansion of haploidentical γδ T cells

**DOI:** 10.1186/1479-5876-12-45

**Published:** 2014-02-15

**Authors:** Martin Wilhelm, Manfred Smetak, Kerstin Schaefer-Eckart, Brigitte Kimmel, Josef Birkmann, Hermann Einsele, Volker Kunzmann

**Affiliations:** 1 Klinikum Nürnberg, Medizinische Klinik 5, Prof-Ernst-Nathan-Str. 1, D-90340 Nuernberg, Germany; 2 Medizinische Klinik und Poliklinik II Wuerzburg, University of Wuerzburg, Wuerzburg, Germany

**Keywords:** Haploidentical γδ T lymphocytes, NK cells, Interleukin-2, Bisphosphonate, Innate immunity, Adoptive transfer, *in vivo* cell expansion

## Abstract

**Background:**

The primary aim of this pilot study was to determine the feasibility and safety of an adoptive transfer and *in vivo* expansion of human haploidentical γδ T lymphocytes.

**Methods:**

Patients with advanced haematological malignancies who are not eligible for allogeneic transplantation received peripheral blood mononuclear cells from half-matched family donors. For that, a single unstimulated leukapheresis product was incubated with both the anti-CD4 and anti-CD8 antibodies conjugated to paramagnetic particles. The depletion procedure was performed on a fully automated CliniMACS® device according to the manufacturer’s instructions. On average, patients received 2.17 × 10^6^/kg (range 0.9-3.48) γδ T cells with <1% CD4- or CD8-positive cells remaining in the product. All patients received prior lymphopenia-inducing chemotherapy (fludarabine 20-25 mg/m^2^ day -6 until day -2 and cyclophosphamide 30-60 mg/kg day -6 and -5) and were treated with 4 mg zoledronate on day 0 and 1.0x10^6^ IU/m^2^ IL-2 on day +1 until day +6 for the induction of γδ T cell proliferation *in vivo*.

**Results:**

This resulted in a marked *in vivo* expansion of donor γδ T cells and, to a lower extent, natural killer cells and double-negative αβ T cells (mean 68-fold, eight-fold, and eight-fold, respectively). Proliferation peaked by around day +8 and donor cells persisted up to 28 days. Although refractory to all prior therapies, three out of four patients achieved a complete remission, which lasted for 8 months in a patient with plasma cell leukaemia. One patient died from an infection 6 weeks after treatment.

**Conclusion:**

This pilot study shows that adoptive transfer and *in vivo* expansion of haploidentical γδ T lymphocytes is feasible and suggests a potential role of these cells in the treatment of haematological diseases.

## Introduction

For many patients with refractory haematological malignancies, allogeneic stem cell transplantation (SCT) remains the only chance of a cure. However, due to its high toxicity, a significant number of patients are not eligible for this approach.

The anti-tumour activity of allogeneic SCT is primarily mediated by an immunological graft-versus-tumour effect mediated by donor lymphocytes. Ruggeri et al. demonstrated that a KIR ligand mismatch enhanced the donor natural killer (NK) cell alloreactivity in haploidentical transplantations, through a “missing self-recognition” in patients with AML [1]. Furthermore, Miller et al. described a successful adoptive transfer and *in vivo* expansion of haploidentical NK cells by interleukin 2 (IL-2) in a non-transplantation setting [2]. The major advantages of immunotherapy with innate lymphocytes compared with MHC-restricted αβ T cells are that they can kill tumour cells without prior exposure and do not induce graft-versus-host disease (GVHD) [3].

However, currently used approaches with allogeneic innate lymphocytes are solely focused on NK cells and underscore the powerful anti-tumour activity of γδ T cells [2,4,5].

Human γδ T cells not only recognise microbial antigens, but are also capable of exerting significant MHC-unrestricted activity against a broad spectrum of tumour cells *in vitro*, especially haematological neoplasia [6,7]. Although their mechanism of target cell recognition is not fully understood, it is known that Vγ9Vδ2 T cells, which represent the vast majority of human circulating γδ T cells, recognise naturally occurring and synthetic phosphoantigens (i.e., aminobisphosphonates such as pamidronate or zoledronate) [8,9].

In two previous trials (phase I/II) in patients with relapsed/refractory malignancies, we demonstrated that a combination of pamidronate or zoledronate and low-dose IL-2 not only induced the selective activation and proliferation of Vγ9Vδ2 T cells *in vivo,* but could also be accompanied by anti-tumour activity [10,11]. However, a general drawback of autologous γδ T cell-mediated tumour-immunotherapy is the frequent impaired function of γδ T cells in up to 50-70% of cancer patients [7,10,12].

Here, we present for the first time data showing that the adoptive transfer of haploidentical γδ T cells is a feasible and safe method for the *in vivo* expansion of these innate immune effector cells in patients with refractory haematological malignancies.

## Methods and materials

### Patients and treatment protocol

In this pilot study, four subsequent patients with advanced refractory haematological malignancies (one T-NHL, one AML, one secondary plasma cell leukaemia, and one multiple myeloma) who were not eligible for allogeneic transplantation were included. HLA typing of the patients and their haploidentical family members were performed to select a donor with an HLA mismatch, which was detectable by FACS analysis with an HLA-specific antibody. Infusion of a CD4/CD8 T cell-depleted leukapheresis product (donor innate lymphocyte infusion = DILI) and Hi-Cy/Flu as prior immunosuppressive chemotherapy (fludarabine 25 mg/m^2^ day -6 until day -2 and cyclophosphamide 60 mg/kg day -6 and -5) were performed as described [2,4,5]. However, according to the general conditions and/or prior therapies, the dose of cyclophosphamide was reduced by 50% in patients 1 and 2, and both cyclophosphamide/fludarabine by 25% in patients 3 and 4. All patients received intravenous (i.v.) zoledronate at a dose of 4 mg after DILI infusion on day 0 and subcutaneous (s.c.) 1.0x10^6^ IU/m^2^ IL-2 on day +1 until day +6.

The collection of PBMCs and depletion of CD4^+^ and CD8^+^ cells were performed as previously described [13]. In brief, a single unstimulated leukapheresis was performed from family donors using a Cobe Spectra (Gambro BCT, Planegg-Martinsried, Germany). The cells were incubated with anti-CD4 and anti-CD8 antibodies conjugated to paramagnetic particles and then processed with the fully automated device CliniMACS® Plus (Miltenyi Biotec) using the program “Depletion 2.1”, according to the manufacturer’s instructions.

This pilot study was approved by the institutional review board and all participants gave written informed consent.

### Flow cytometric analysis

Leukapheresis products and patient samples were analysed using CD3, CD4, CD8, CD14, CD15, CD16 + CD56, CD19, TCRαβ, and TCRγδ fluorochrome-labelled antibodies by four-colour FACS analysis (Epics XL/FC500, Beckman Coulter). *In vivo* expansion of HLA-mismatched donor lymphocytes was followed by FACS-based chimerism analysis with prior tested specific antibodies every other day.

## Results and discussion

**Figure 1:**
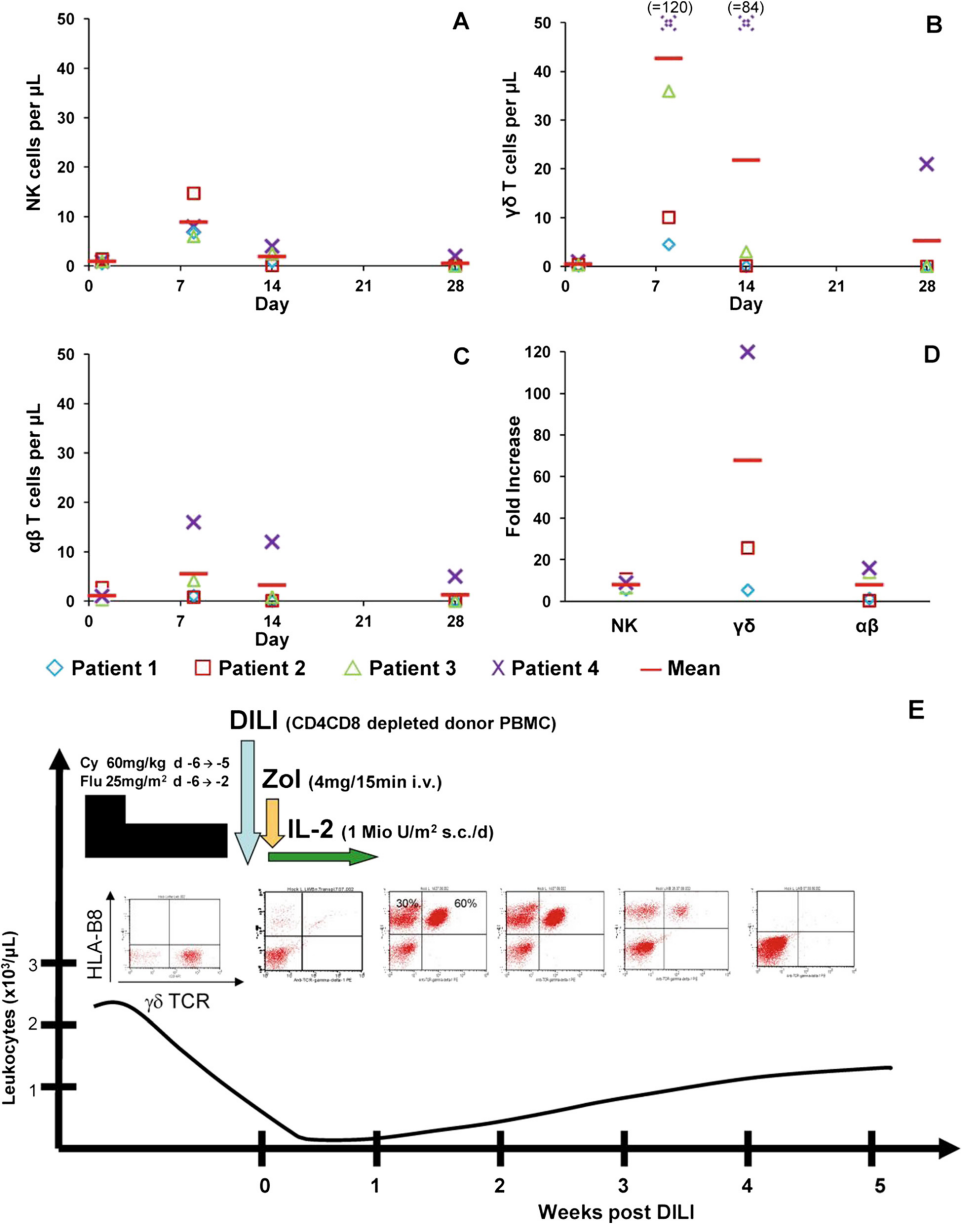
**Treatment protocol and cell engraftment.** Number of donor natural killer (NK) cells **(A)**, γδ T cells **(B)**, and αβ T cells **(C)** per microlitre. The individual symbols represent patient samples obtained at the different time points. Peripheral blood lymphocytes were analysed using fluorochrome-labelled TCRαβ, TCRγδ, CD56, and donor-specific HLA antibodies by four-colour FACS analysis. **(D)** Fold increase is calculated as the ratio of the cell number per microlitre on day 8 to the initial value. **(E)** Treatment and adoptive transfer of donor innate lymphocytes in patient 3, who had a refractory plasma cell leukaemia. *In vivo* expansion of HLA-B8^+^ haploidentical γδ T cells within the HLA-B8^-^ recipient was followed by FACS-based chimerism analysis. Right upper quadrant shows the percentage of donor γδ T cells over time.

The four subsequent patients with advanced refractory haematological malignancies received their leukapheresis product from unstimulated haploidentical family donors in the lymphopenic phase after prior cyclophosphamide/fludarabine treatment (Figure 1E). Preservation of the function of γδ T cells was tested after separation by proliferation and cytotoxicity assays against different targets (data not shown) [13]. The product contained 2.17 × 10^6^/kg (range 0.9-3.48) γδ T cells and 8.87 × 10^6^/kg (range 1.54-18.82) NK cells (Table 1). Although a significant number of αβ T cells remained in the transplant (0.7-2.06 × 10^6^/kg), this fraction was primarily composed of CD4- and CD8-negative cells (Table 1). Double-negative (DN) T cells have been shown to possess the ability to regulate immune responses in transplant rejection, tumour immunity, infectious diseases, and GVHD [14-16]. Therefore, our separation products, engineered by CD4/CD8 depletion, were supplemented by a subpopulation of αβ T cells, which have further interesting capabilities regarding the prevention of GVHD and tumour immunity.

**Table 1:** Patient characteristics and cell products

	**Patient 1**	**Patient 2**	**Patient 3**	**Patient 4**
**Patient characteristics**				
Age/sex	44/m	69/m	70/m	65/m
Disease	T-NHL	AML	SPL	MM
No. prior regimens	4	1	3	7
Donor	sister	son	daughter	son
Best response	CR	CR	CR	n.e.
a/c GVHD	-/-	-/n.e.	-/-	-/n.e.
Outcome	R/5 mo	R/2 mo	R/8 mo	e.d./6 wk
**Cell dose (10**^**6**^**/kg)**				
γδ T cells	2.98	0.99	1.23	3.48
NK cells	18.82	12.85	2.26	1.54
αβ T cells	1.74	1.14	2.06	0.70
CD4^+^ T cells	0.01	0.03	0.02	0.006
CD8^+^ T cells	0.04	0.06	0.004	0.02
Total	23.59	15.07	5.57	5.75
KIR-mm	+	-	+	+

T-NHL indicates T-cell Non-Hodgkin-Lymphoma; AML, Acute Myeloid Leukaemia; SPL, Secondary Plasma Cell Leukaemia; MM, Multiple Myeloma; a/c GVHD, acute/chronic graft-versus-host disease; R, relapse; n.e., not evaluable; e.d., early death; KIR-mm, KIR ligand incompatibility in the graft-versus-host direction.

### Engraftment and expansion of donor cells

Our prior trials in the autologous setting clearly showed that the expansion of γδ T cells *in vivo* was a prerequisite for tumour regression, since only patients with significant *in vivo* proliferation of γδ T cells exhibited a tumour response [10]. Therefore, one major goal of our study was to investigate the feasibility and tolerability of selective *in vivo* expansion of the adoptively transferred haploidentical γδ T cells. For that, patients received a phosphoantigen (4 mg zoledronate) and low-dose IL-2 (1-2 × 10^6^ IE) s.c. for 6 days, starting on day 1 after transfusion. The results showed that a transient but significant expansion of donor γδ T cells and, to a lower extent, of donor NK cells and DN αβ T cells occurred in these patients (Figure 1). In all patients, proliferation peaked by around day +8, while on day +15, in three out of four patients there were no detectable donor cells, concurrent with the regeneration of recipient cells. The mean number of γδ T cells, NK cells and DN αβ T cells was approximately 43 cells/μL, 9 cells/μL, and 6 cells/μL, which represented a 68-fold, an eight-fold, and an eight-fold expansion, respectively. However, in patient 4, who showed the most profound γδ T cell expansion, cells were detectable for as long as 28 days after transfusion. Due to the underlying disease and multiple prior therapies including autologous stem cell transplantation, patient 4 was severely immunosuppressed, suggesting that the degree of immunosuppression may be a determinant in the duration of engraftment. Miller et al. have already shown that the survival of haploidentical lymphocytes is dependent on the extent of prior immunosuppression by comparing low- and high-intensity regimens [2].

### Side effects

The duration of neutropenia (< 500/μl) was 20 (range 11-33) days. Although it has not been shown that γδ T cells recognise alloantigens, potential hazardous effects of *in vivo* stimulated mismatched γδ T cells was of major concern. Although patient 4 died from severe septicaemia (*Staphylococcus epidermidis + Enterobacter faecium*) on day +45, with his haematopoiesis already recovered, none of the patients showed any signs of acute or chronic GVHD nor organ injury (Table 1).

### Efficacy

Three out of four patients achieved a complete remission, which lasted between 2 and 8 months. For example, patient 3, who suffered from secondary plasma cell leukaemia and did not respond to 3 different regimens including autologous stem cell transplantation, also achieved a stringent complete remission (Figure 1).

It cannot be excluded, that the Flu/Cy regimen given prior cell transfusion for lymphopenia induction, induced a significant tumour regression in the patients. However, refractoriness to all other prior treatment approaches and the kind of underlying diseases argues against this assumption. Due to the small patient number, we were not able to differentiate which cell population was the main contributor for remission induction. Figure 1 shows the expansion of NK cells, αβ T cells and γδ T cells. It is very likely that the αβ T cell population consists at least in part of double-negative (classically Va24 + CD3+) invariant natural killer T (iNKT) cells. Given the known efficacy of iNKT cells against lymphoid tumour cells, it is possible that besides αβ T cells and “classical” NK cells, this subpopulation contributed to the anti-tumour effect, particularly in the cases of lymphoid malignancies [14,17-20].

## Conclusion

This pilot study showed for the first time that infusion of haploidentical γδ T cells is feasible and the administration of phosphoantigens and low-dose IL-2 induces a significant proliferation of donor γδ T cells *in vivo*[10,11]. In addition, this clinical trial provides the first evidence that selective stimulation of allogeneic γδ T cells can be accompanied by anti-tumour activity *in vivo* without inducing GVHD.

## Competing interests

M.W. and V.K. were supported by a grant from Miltenyi Biotec GmbH, Bergisch Gladbach, Germany.

## Authors’ contributions

MW wrote the paper; MW and VK designed the study and interpreted the data; MS and BK collected and analysed data and edited the paper; KSE and JB performed the study; HE edited the paper. All authors approved the final version of the manuscript.
